# Internal Ribosome Entry Sites Mediate Cap-Independent Translation of Bmi1 in Nasopharyngeal Carcinoma

**DOI:** 10.3389/fonc.2020.01678

**Published:** 2020-09-08

**Authors:** Hongbo Wang, Yunjia Zhu, Lijuan Hu, Yangyang Li, Guihong Liu, Tianliang Xia, Dan Xiong, Yiling Luo, Binliu Liu, Yu An, Manzhi Li, Yuehua Huang, Qian Zhong, Musheng Zeng

**Affiliations:** ^1^Guangdong Provincial Key Laboratory of Liver Disease Research, The Third Affiliated Hospital of Sun Yat-sen University, Guangzhou, China; ^2^State Key Laboratory of Oncology in South China, Collaborative Innovation Center for Cancer Medicine, Sun Yat-sen University Cancer Center, Guangzhou, China; ^3^Beijing Key Laboratory of Hematopoietic Stem Cell Transplantation, Peking University People’s Hospital, Peking University Institute of Hematology, Beijing, China; ^4^Department of Pathology, Sun Yat-sen Memorial Hospital, Guangzhou, China; ^5^Tungwah Hospital of Sun Yat-sen University, Dongguan, China; ^6^Department of Laboratory Medicine, Luohu District People’s Hospital, Shenzhen, China

**Keywords:** internal ribosome entry sites, Bmi1, nasopharyngeal carcinoma, 5′-untranslated region, translational activity

## Abstract

Bmi1 is overexpressed in multiple human cancers. We previously reported the oncogenic function and the transcription regulation mechanisms of Bmi1 in nasopharyngeal carcinoma (NPC). In this study, we observed that the mRNA and the protein levels of Bmi1 were strictly inconsistent in NPC cell lines and cancer tissues. The inhibitors of proteasome and lysosome could not enhance the protein level of Bmi1, indicating that Bmi1 may be post-transcriptionally regulated. The IRESite analysis showed that there were two potential internal ribosome entry sites (IRESs) in the 5′-untranslated region (5′-UTR) of Bmi1. The luciferase assay demonstrated that the 5′-UTR of Bmi1 has IRES activity, which may mediate cap-independent translation. The IRES activity of the Bmi1 5′-UTR was significantly reduced after the mutation of the two IRES elements. Taken together, these results suggested that the IRES elements mediating translation is a novel post-transcriptional regulation mechanism of Bmi1.

## Highlights

- We observed that the mRNA and the protein levels of Bmi1 were strictly inconsistent.

- The inhibitors of proteasome and lysosome could not enhance the protein level of Bmi1.

- The 5′-UTR of Bmi1 mRNA contains potential internal ribosome entry sites.

- The 5′-UTR of Bmi1 mediated cap-independent translation.

- The mutation of the two IRES elements reduced the IRES activity of the Bmi1 5′-UTR.

## Introduction

Nasopharyngeal carcinoma (NPC) is a common malignant tumor in Southern China and Southeast Asia. Chemotherapy and radiation therapy are the preferred options for the treatment of NPC. However, the 5-year survival rate is only 50 to 75% (1). Therefore, it is very important to elucidate the molecular mechanism underlying the progression of NPC.

Bmi1, a member of the polycomb-repressive complex 1 (PRC1), participates in the self-renewal of hematopoietic and neural stem cells, embryogenesis, cell cycle regulation, senescence, chemoresistance, recurrence of cancer stem cells, and tumor progression (2, 3). Bmi1 is upregulated in tumor cells and tissue and associated with poor prognosis in various human cancers including breast cancer (4), lymphomas (5), melanoma (6), colon cancer (7), ovarian cancer (8), hepatocellular carcinoma (9), NPC (10, 11), lung cancer (12), and neuroblastoma (13). We previously reported that the over-expression of Bmi1 promoted immortalization and epithelial–mesenchymal transition (EMT) in nasopharyngeal epithelial cells (11, 14). The knockdown of Bmi1 reverses EMT and suppresses the metastasis of NPC cells. Xu et al. reported that the knockdown of Bmi1 sensitizes CD44^+^ nasopharyngeal cancer stem-like cells to radiotherapy (15). These findings indicate that Bmi1 may serve as a potential therapeutic target for NPC (16).

The overexpression of Bmi1 in cancers could be caused by different mechanisms. We previously reported that Sp1 and c-Myc regulate the transcription of Bmi1 mRNA in NPC (17). Nowak reported that E2F1 and MYCN promote the transcription of Bmi1 in neuroblastomas (18). Hypoxia-induced Twist1 directly enhances Bmi1 transcription and cooperates with Bmi1 to induce EMT and stemness properties in head and neck squamous cell carcinoma (19). SALL4 and c-Myb regulates the transcription of Bmi1 in hematopoietic and leukemic cells, while nuclear factor kB (NF-kB) and the sonic hedgehog-activated Gli1 are its potent transcription factors in Hodgkin lymphoma and medulloblastoma cells, respectively (20–23). In addition to transcriptional regulation, miR-200C (24), miR-141 (25), miR-203 (26), miR-15a (27), miR-183 (26), miR-218 (28), miR-320a (10), and miR-16 (27) target Bmi1 and suppress its expression. Moreover, Bmi1 expression may also be regulated post-translationally. BetaTrCP promotes the ubiquitination and the degradation of Bmi1 protein in breast cancer cells (29). C18Y polymorphism in the RING finger domain of Bmi1 promotes its degradation through the ubiquitin–proteasome system (30). The PS domain of Bmi1 is involved in its proteolysis, and it negatively regulates the function of Bmi1 oncoprotein (31). Although there are mountains of studies addressing the regulation of Bmi1 in the transcriptional, post-transcriptional, and post-translational levels, there are few studies about the role of internal ribosome entry site (IRES) elements in the regulation of Bmi1 expression.

Protein biosynthesis includes cap-dependent and cap-independent initiations, which are directed by IRES. Since the discovery of the IRES elements in the 5′-untranslated regions (5′-UTRs) of the encephalomyocarditis virus (32) and the poliovirus (33), IRESs have been found to be contained in the 5′-UTR of a number of viral and eukaryotic cellular mRNAs, including proto-oncogenes, growth factors, receptors, and transcription factors (34). Cellular mRNA with IRES elements could regulate angiogenesis, mitosis, various stress situations, and nutritional and osmotic control (35). IRES *trans*-acting factors (ITAFs) are RNA binding proteins which are required for IRES-mediated translation and thus specifically enhance the protein level of mRNA with IRES elements. PTBP1 (36), PCBP1 (37), HnRNP A1 (38, 39), HnRNP C (40), and DAP5 (41) are the reported ITAFs which regulate the translation of numerous oncogenes, such as MYC, FGF2, IGF1R, CDK1, cyclin D1, etc.

In the present study, we reported that there are two IRES motifs in the 5′-UTR of the human Bmi1. The mutation of the two IRES elements partially inhibited the translational activity of the Bmi1 5′-UTR. These results demonstrated that IRES elements might mediate the translation of Bmi1 in NPC.

## Materials and Methods

### Materials

Proteasome inhibitor MG132 (Calbiochem, San Diego, CA, United States) was used at a final concentration of 10 and 20 μM for 8 h. The cell was treated with lysosomal inhibitor NH_4_Cl (Guangzhou Chemical Reagent Factory, China) at a final concentration of 10 and 20 mM for 8 h. The β-gal was purchased from Sigma.

### Cell Culture

HNE1, CNE2, and C666-1 cells (the human nasopharyngeal carcinoma cells) were grown in RPMI1640 medium, supplemented with 10% fetal bovine serum (Gibco, Grand Island, NY, United States). MCF10A cells (the immortalized primary human mammary epithelial cells) were maintained in keratinocyte/serum-free medium (Gibco). MCF7 cells (the human breast cancer cells) were grown in Dulbecco’s modified Eagle’s medium (Gibco) supplemented with 10% fetal bovine serum (Gibco). The cells were grown in a humidified 5%-CO_2_ incubator at 37°C and passaged with standard cell culture techniques.

### Patients and Specimens

Four paraffin-embedded slides and the paired mRNA from NPC specimens were obtained from Sun Yat-sen University Cancer Center (17). Informed consent was obtained from each patient prior to surgery, and the study was approved by the Institute Research Ethics Committee of Sun Yat-sen University Cancer Center.

### Immunohistochemistry

An immunohistochemical analysis of Bmi1 expression in NPC specimens was performed as previously described (11). Each slide was incubated overnight with rabbit antibody against human Bmi1 (Proteintech Group, Wuhan, China).

### Plasmid Constructs

The plasmid (pRBMIF) was constructed by the insertion of the 5′-UTR of Bmi1 (GenBank accession number: NM_005180.6) PCR-amplified from the cDNA of C666-1 into the *Eco*RI/*Nco*I sites between the Renilla and the Firefly luciferase in the dual luciferase vector pRF (42) (a generous gift from Dr. Anne Willis, University of Leicester, United Kingdom). The 5′-UTR of Bmi1 containing mutant IRES1, IRES2, or both was obtained through the overlap extension PCR method, with pRBMIF as the template, and inserted in the plasmid (pRF), named as mIRES1, mIRES2, and mIRES1 + 2, respectively. The primers used in the overlap extension PCR were as follows: mutant IRES1, 5′-CCA CCATCGCATCGCACGCTTCGCATCGCCCCGCTCGCACGC ACACACACGGC-3′ and mutant IRES2, 5′-CGTCATGCACC GGGGGCTGCTTCGCATCGTGCTCGGCCGCCGCCGCCTC CTCCCGCTCC-3′.

### Western Blotting Analysis

Western blotting analysis was performed as previously described (43). The cells were harvested and lysed with a cell lysis buffer [50 mM Tris–HCl (pH 7.4), 150 mM NaCl, 1.0% NP-40, 5 mM EDTA, and protein inhibitor cocktail]. The cell lysates were subjected to SDS-PAGE and immunoblotted with Bmi1 (Proteintech Group, Wuhan, China), α-tubulin (Sigma), glyceraldehyde-3-phosphate dehydrogenase (GAPDH; Santa Cruz Biotechnology, Santa Cruz, CA, United States), and p53 (Santa Cruz Biotechnology) as indicated.

### Transient Transfections and IRES Activity Assays

CNE2 (1 × 10^5^) cells were seeded into 24-well plates 16 h prior to transfection, at about 70–80% confluency. The cells were co-transfected with 200 ng of the indicated dicistronic reporter plasmids and 100 ng of β-galactosidase reporter plasmid using Lipofectamine 2000 (Invitrogen) according to the manufacturer’s protocol. β-Galactosidase reporter plasmid serves as an internal control to correct the differences in both the transfection and the harvesting efficiencies. At 24 h following transfection, cell extracts were harvested using a passive lysis buffer (Promega, Madison, WI, United States) and then subjected to the translational activity assays. The Firefly and the Renilla luciferase activities were determined using the dual-luciferase reporter assay system (Promega). The β-galactosidase activity was analyzed with the galactosidase enzyme assay system (44). The translational activity was determined as the ratios of Firefly to Renilla luciferase activities relative to the value of the β-galactosidase activity.

### RT-PCR and Quantitative RT-PCR Analysis

Total RNA was extracted with TRIzol reagent (Invitrogen) as described previously (17). According to the manufacturer’s instructions, the first-strand cDNA was synthesized using 2 μg of the total RNA with a reverse transcriptase kit (Promega). The total RNA was treated with DNAseI for the Firefly and the Renilla luciferase gene-transfected cell and qRT-PCR analysis. The mRNA level was evaluated by qRT-PCR with Power SYBR Green qPCR SuperMix-UDG (Invitrogen) on an LC480 detection system (Roche). GAPDH was used as an internal control to normalize the relative expression. The relative gene expression was calculated by 2^ΔCt^, where ΔCt = Ct (internal control) – Ct (unknown). The following primers were used in the qRT-PCR analysis: qBmi1pF, 5′-TGGCTCGCATTC ATTTTCTG-3′; qBmi1pR, 5′-TGTGGCATCAATGAAGT ACCCT-3′; qGAPDHpF, 5′-CTCCTCCTGTTCGACAGT CAGC-3′; qGAPDHpR, 5′-CCCAATACGACCAAATCC GTT-3′; qFireflypF, 5′-CGGATTACCAGGGATTTCA GTC-3′; qFireflypR, 5′-ATCTCACGCAGGCAGTTC TATG-3′; qRenillapF, 5′-GCCTCGTGAAATCCCGTTAGT-3′; and qRenillapR, 5′-TCTTGGCACCTTCAACAATAGC-3′.

The full-length 5′-UTR of Bmi1 was 623 bp and was amplified using specific primers (BMI-UTRpF: 5′-GGAA TTCGAGCCATTTTGGAGCCGGTG-3′ and BMI-UTRpR: 5′-CATGCCATGG TTCTGCTTGATAAAAAATCCCGG-3′). The internal reference gene was ACTIN (ACTINpF: 5′-CGCGAGAA GATGACCCAGAT-3′ and ACTINpR: 5′-GGGCATACCC CTCGTAGATG-3′), and the final product was about 500 bp long. These products were analyzed by electrophoresis on 1.0% agarose gel.

### IRES and RNAfold Analyses

The IRESite^1^ presents curated experimental evidence of many eukaryotic viral and cellular IRES regions (45). RNAfold^2^ is the web-based algorithm which was used for the predictions of the RNA secondary structure.

### Statistical Analysis

All data analyses were conducted with GraphPad Prism version 5.0 (GraphPad Software, San Diego, CA, United States). The results were representative of at least three independent experiments.

Data were presented as mean ± standard error of the mean obtained with triplicate samples. The analysis of the differences between groups was determined with a *t*-test, and differences with *p* < 0.05 were considered as statistically significant.

## Results

### The mRNA and the Protein Levels of Bmi1 Were Inconsistent in Nasopharyngeal Carcinoma Cancer Cells and Cancer Tissues

**FIGURE 1:**
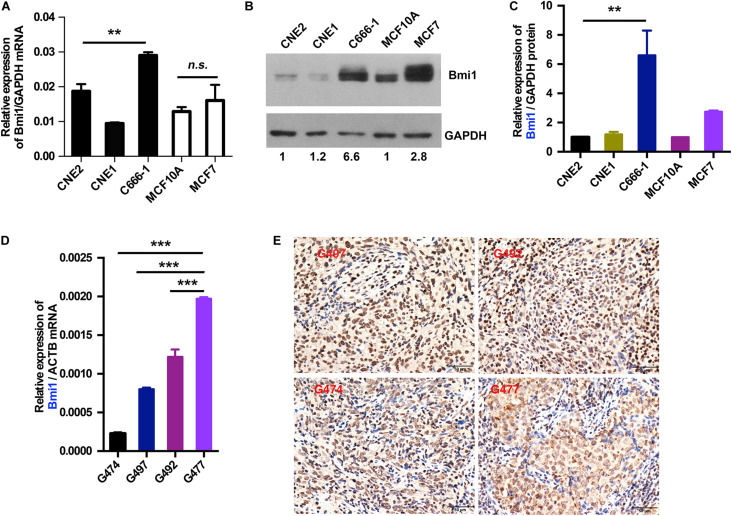
The mRNA and protein levels of Bmi1 are inconsistent in nasopharyngeal carcinoma, breast cancer cell lines, and nasopharyngeal carcinoma tissues. **(A,B)** The protein and mRNA levels of Bmi1 were analyzed by qRT-PCR **(A)** and Western blot **(B)** in CNE2, C666-1, MCF10A, and MCF7 cell lines. GAPDH was used as internal control. **(C)** The intensity of the bands was determined with ImageJ software. **(D,E)** The mRNA and protein levels of Bmi1 were analyzed by qRT-PCR **(D)** and immunohistochemistry staining **(E)** in nasopharyngeal carcinoma tissues. *ACTB* was used as qPCR internal control. A representative result of three independent experiments is shown (*n* = 3). The graphs show the mean ± SEM. **P < 0.01, ***P < 0.001; Student’s *t*-test.

To explore the mechanism underlying the high expression of Bmi1, the mRNA and the protein levels of Bmi1 in NPC and breast cancer cells were determined. As shown in Figure 1, the expression of Bmi1 mRNA in C666-1 cells was only 1.5-fold higher than in CNE2 cells (Figure 1A). However, the Bmi1 protein level in C666-1 cells was 6.6-folds higher than in CNE2 cells (Figures 1B,C). In addition to NPC cells, the protein level in MCF7 cell was 2.8-folds higher than in MCF10A cells, while Bmi1 mRNA was almost equal in MCF10A and MCF7 cells (Figures 1A–C).

To clinically show the inconsistency between the mRNA and the protein levels of Bmi1, we analyzed the expression of Bmi1 mRNA and protein in NPC tissue. As shown in Figures 1D,E, the expression of Bmi1 mRNA in G477 NPC tissue was higher than in G474, G497, and G492. However, immunohistochemical staining showed that the Bmi1 protein level in G474, G497, and G492 was higher than in G477 NPC tissue.

Taken together, the mRNA and the protein levels of Bmi1 were not strictly correlated in NPC cancer cells and tissues, indicating that Bmi1 expression was not only regulated in the transcriptional level but may also be regulated in the post-transcriptional level in NPC cells and cancer specimens.

### Inhibitors of Proteasome and Lysosome Could Not Enhance the Protein Level of Bmi1

**FIGURE 2:**
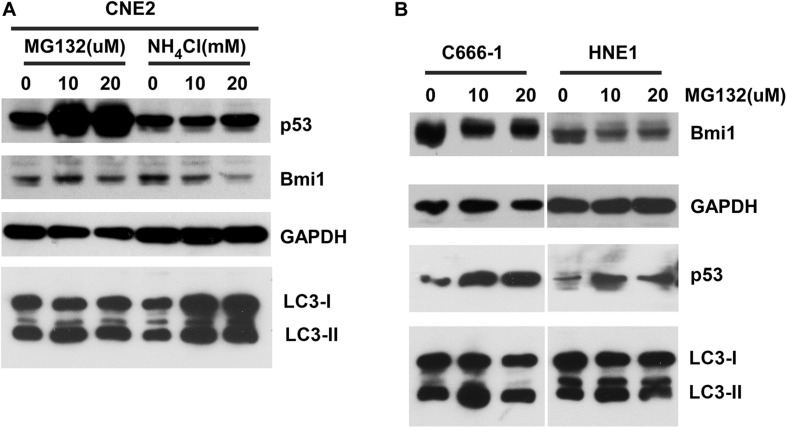
The inhibitors proteasome and lysosome do not have effects on Bmi1 protein expression in nasopharyngeal carcinoma (NPC) cells. NPC cells CNE2 **(A)**, C666-1, and HNE1 **(B)** were seeded into six-well plates, then treated with the indicated concentrations of MG132 and NH_4_Cl for 8 h, then subjected to western blot. p53 served as the positive control for the role of MG132 in proteasomal degradation. LC3-II served as the positive control for the role of NH_4_Cl in lysosomal degradation. GAPDH was used as a loading control. The data represent three sets of independent experiments.

To determine whether Bmi1 was regulated at the posttranslational level in NPC cell lines, the effects of proteasomal inhibitors (MG132), and lysosomal inhibitors (NH_4_Cl) on the protein level of Bmi1 were analyzed. CNE2 cells were cultured in the presence of the indicated concentration of MG132 and NH_4_Cl for 8 h. As shown in Figure 2A, compared to the DMSO-treated cells, MG132 and NH_4_Cl obviously enhanced the protein level of p53 (the positive control for proteasomal degradation) and LC3-II (the positive control for lysosomal degradation), respectively. However, both MG132 and NH_4_Cl could not enhance the protein level of Bmi1 in CNE2 cells. In addition, the NPC cell lines HNE1 and C666-1 were also treated with MG132 for 8 h. The protein level of Bmi1 was not enriched, while the protein level of p53 was obviously increased in MG132-treated cells (Figure 2B). These data indicated that the expression of Bmi1 might be post-transcriptionally regulated in the proteasome-independent and lysosome-independent degradation pathway.

### Two Internal Ribosome Entry Sites Are Identified in Bmi1 5′-UTR

**FIGURE 3:**
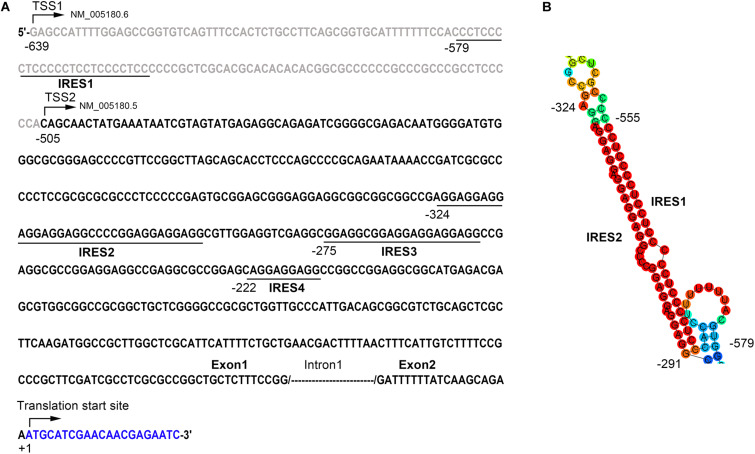
The 5′-UTR region of Bmi1 contains the potential internal ribosome entry sites (IRES) motifs. **(A)** IRESite database analyses of the region from −639 to +1 of Bmi1 5′-UTR sequence; the translation initiation site ATG was assigned as +1. There are four potential IRES elements. **(B)** The secondary structure of the Bmi1 5′-UTR predicated by RNAfold database showing that the IRES1 and IRES2 motifs form a KMI1 stem loop structure.

To further explore the potential mechanisms underlying the inconsistency between the mRNA and the protein level of Bmi1, we analyzed the region from −639 to +1 (the translation initiation site ATG was assigned as +1) of the Bmi1 5′-UTR using the IRESite online software. As shown in Figure 3A, there were four potential IRES motifs, which were named as IRES1 (−579/−549), IRES2 (−324/−291), IRES3 (−275/−255), and IRES4 (−222/−213). The RNAfold online software was used to analyze the secondary structure of the Bmi1 5′-UTR. As shown in Figure 3B, the IRES1 and IRES2 motif in the 5′-UTR region of Bmi1 could form the putative KMI1 stem loop structure which may bind PTBP1. These results suggested that the two potential IRES motifs in the Bmi1 5′-UTR may activate the translation of Bmi1.

### The Bmi1 5′-UTR Promoted Translation

**FIGURE 4:**
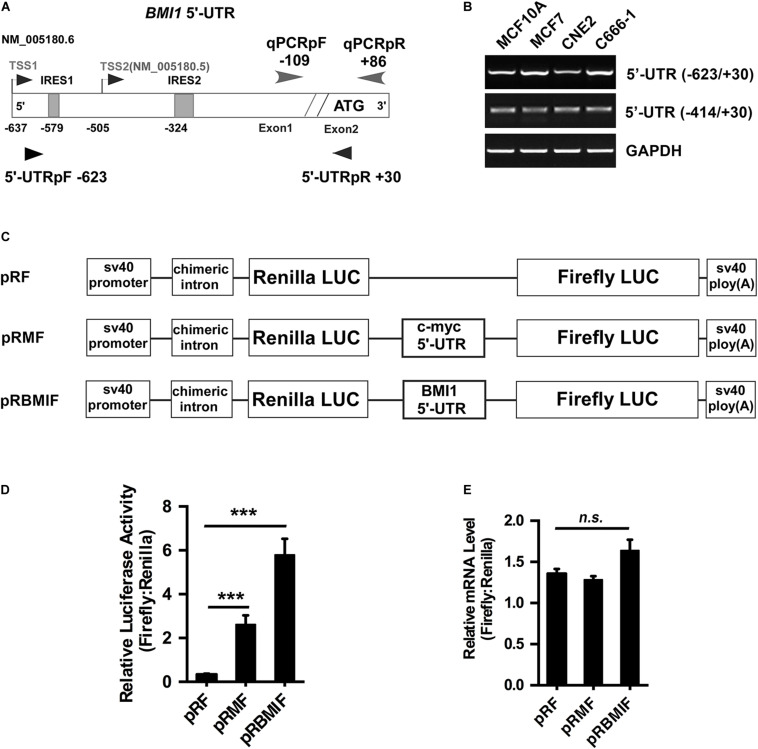
The 5′-UTR promotes the translation of Bmi1 in CNE2 cells. **(A)** Diagram of the full-length Bmi1 5′-UTR. **(B)** The Bmi1 5′-UTR in CNE2, C666-1, MCF7, and MCF10A cells was analyzed by PCR. **(C)** Construction of the dicistronic reporter plasmid. The 5′-UTRs of c-myc and Bmi1 were subcloned between the Renilla and the Firefly luciferase in the dual luciferase vector pRF, named as pRMF and pRBMIF, respectively. The plasmid pRMF served as the positive control. **(D)** The translational activity analysis of the 5′-UTR in CNE2 cells. The plasmids pRF, pRMF, and pRBMIF were transfected into CNE2 cells. Luciferase activity was detected 24 h post-transfection and presented as relative internal ribosome entry sites activity (Firefly luciferase/Renilla luciferase). **(E)** The mRNA expression ratio of Firefly to Renilla luciferase in CNE2 cells. A representative result of three independent experiments is shown (*n* = 3). The graphs show the mean ± SEM. ***P < 0.001; Student’s *t*-test.

There are two transcriptional versions of Bmi1 mRNA (NM 005180.6 and NM 005180.5) with different transcription start sites (TSS). The long 5′-UTR (−623/+30) was named as TSS1 and the short 5′-UTR (−414/+30) was named as TSS2 (Figure 4A). RT-PCR demonstrated that both the long and the short 5′-UTRs were expressed in the four cell lines tested, including CNE2, C666-1, MCF10A, and MCF7 cells (Figure 4B).

To test whether the Bmi1 5′-UTR could enhance Bmi1 protein translation, the dicistronic reporter plasmid (named as pRBMIF) was constructed. As shown in Figure 4C, the Bmi1 5′-UTR (−623/+1) was inserted between the Renilla and the Firefly luciferase in the plasmid (pRF), with the 5′-UTR of c-myc as the positive control (pRMF). The plasmids pRF, pRBMI1F, and pRMF were transfected into CNE2 cell lines. As shown in Figure 4D, the luciferase activity of Firefly compared to that of Renilla is 20-folds higher in pRBMIF-transfected cells than in pRF-transfected cells. However, there is no significant difference in the ratio of the mRNA level of Firefly to that of Renilla luciferase in CNE2 cells (Figure 4E). These results suggested that the Bmi1 5′-UTR can effectively activate Bmi1 translation.

### IRES1 and IRES2 Mediate the Translational Activity of the Bmi1 5′-UTR

**FIGURE 5:**
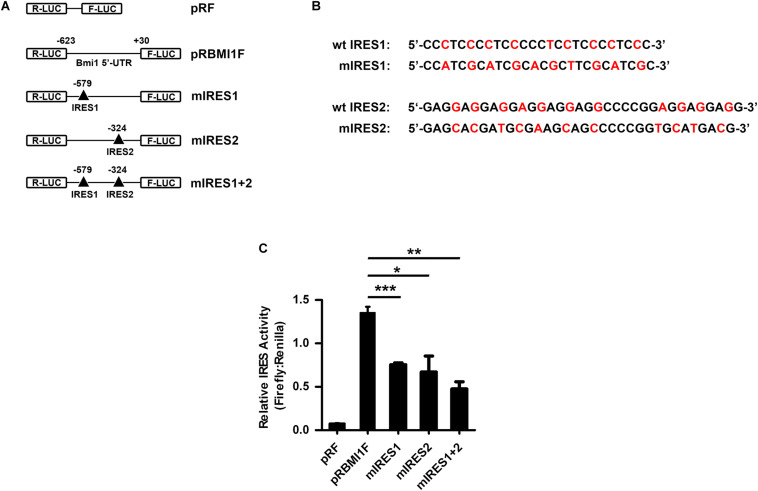
IRES1 and IRES2 mediate the translational activity of 5′-UTR in CNE2 cells. **(A)** Schematic of the potential IRES1 and IRES2 elements in the Bmi1 5′-UTR. The mutation is labeled as “Δ.” The IRES1 and IRES2 or double mutations of Bmi1 5′-UTR were subcloned in pRF vector and were designated as mut-IRES1, mut-IRES2, and mut-IRES1 + 2. **(B)** The IRES1 and IRES2 mutant base was labeled as red. **(C)** The translational activity analysis of the IRES 1 and IRES2 mutant 5′-UTR of Bmi1 in CNE2 cells. The plasmids containing the IRES1 and IRES2 mutant 5′-UTR were transfected into CNE2 cells. Luciferase activity was detected at 24 h post-transfection. A representative result of three independent experiments is shown (*n* = 3). The graphs show the mean ± SEM. *P < 0.05, **P < 0.01, and ***P < 0.001; Student’s *t*-test.

To investigate whether IRES1 and IRES2 regulate the 5′-UTR mediating Bmi1 translation, IRES1 and IRES2 were point-mutated (**F**igures 5A,B). The mutants of IRES1 and IRES2 were cloned into the pRF plasmid and named as mut-IRES1 and mut-IRES2, respectively. The double mutants of both IRES1 and IRES2 were also constructed and named as mut-IRES1 + 2. These plasmids were transfected into CNE2 cells for 24 h, followed by luciferase assays. As shown in Figure 5C, compared to the wild-type control (pRBMIF), the translational activity of mIRES1 and mIRES2 significantly decreased. In addition, double mutations of IRES1 and IRES2 led to an additional decrease in the translational activity, suggesting that both IRES1 and IRES2 were required for the translational activity of the Bmi1 5′-UTR.

## Discussion

Bmi1, a component of PRC1, has been implicated in the tumorigenesis of multiple tumors. It is over-expressed and correlated with a poor prognosis in NPC. However, the mechanism underlying its higher expression remains to be investigated. In this study, we found that the mRNA and the protein level of Bmi1 were not strictly consistent in NPC cancer cells and tissues. We then identified two IRES elements which played an important role in the translational activity of the Bmi1 5′ UTR using the dicistronic reporter plasmid assay. These results elucidated a novel post-transcriptional mechanism of Bmi1 (Figure 1).

We previously identified the region from +167 to +232 and the region from −536 to −134 as the core promoter regions of the Bmi1 gene. The transcriptional activity of the Bmi1 promoter is mainly mediated by the Sp1 binding site cluster (+181/+214) and the E-box elements (−181). It is interesting that the Sp1 binding site cluster (+181/+214, the NM 005180.5 transcription start site was assigned as +1) and IRES2 (−324/−291, the translation initiation site ATG was assigned as +1) are the same sites, suggesting that this region may regulate both the transcription and the translation of Bmi1 in NPC cells (17). With the relative transcriptional activity of IRES2, its cryptic promoter activity does not make a significant contribution to Firefly luciferase expression in Bmi1 5′-UTR (Figure 4E). To understand the significance of the IRES elements in the regulation of Bmi1 translational activity, the IRES1 and IRES2 motifs were truncated. The translational activity of the IRES mutants was significantly reduced but did not abrogate (Figure 5), suggesting that there are additional potential IRESs in the Bmi1 5′-UTR.

To investigate whether there are mutations of IRES1 and IRES2 in the Bmi1 5′UTR, the RNA-seq data from 113 patients with NPC (GSE102349, https://www.ncbi.nlm.nih.gov/geo/query/acc.cgi?acc=GSE102349) were analyzed for the mutation of IRES2 in the Bmi1 5′-UTR. The G/A mutation (GGAG GAGGAGGAGGAGGCCCCGGAGGAGGAGG → AGAGG AGGAGGAGGAGGCCCCGGAGGAGGAGG) was found in the IRES2 motif of the sample (SRR5908825). No mutation was found in the IRES2 motif of the rest of the 112 samples (Supplementary Figure 1). It is hard to analyze the mutation in the IRES1 element, which locates nearby the transcription start sites of Bmi1. The data suggested that the mutation of BMI1 IRES is a rare event.

In addition to the IRES element, the ITAFs are required for IRES-mediated cap-independent translation. The expression level of the ITAFs may also affect the translation of Bmi1. Therefore, it is very important to further identify ITAFs and analyze their expression levels in cancer patients. IRES1 and IRES2 form the stem loop structure of KMI1, which could bind to PTBP1 (46). To identify whether PTBP1 regulated the translational activity of the Bmi1 5′ UTR, we assessed the effect of PTBP1 knockdown on Bmi1 expression. Both the mRNA level and the protein level of Bmi1 did not change in the PTBP1-knockdown CNE2 cells. Therefore, it is important to further identify the ITAFs which may regulate the translational activity of Bmi1.

In conclusion, the IRES motifs in the 5′-UTR regulated the translation of Bmi1. The ITAFs–Bmi1 pathway may play an important role in the development of NPC and other tumors. It is urgent to explore the ITAFs that bind to Bmi1 IRES elements and thus regulate the translation of Bmi1. The identification of ITAFs would provide a potential therapeutic target for NPC treatment.

## Data Availability Statement

All datasets generated for this study are included in the article/Supplementary Material. The authenticity of this article has been validated by uploading the key raw data onto the Research Data Deposit public platform (www.researchdata.org.cn), with the approval number RDDB2020000951.

## Ethics Statement

The studies involving human participants were reviewed and approved by The Institute Research Ethics Committee of Sun Yat-sen University Cancer Center. The patients/participants provided their written informed consent to participate in this study.

## Author Contributions

MZ, HW, and QZ designed and oversaw the project. HW, YZ, LH, and GL performed the key experiments, analyzed the data, and wrote the manuscript. YiL and DX performed the cell culture and western blotting. BL and YaL performed the immunohistochemistry assays. TX performed the bioinformatic analysis. YH, ML, and YA provided technical support and important materials. All the authors read and approved the final manuscript.

## Conflict of Interest

The authors declare that the research was conducted in the absence of any commercial or financial relationships that could be construed as a potential conflict of interest.

## Footnotes

1 http://www.iresite.org

2 http://nhjy.hzau.edu.cn/kech/swxxx/jakj/dianzi/Bioinf4/miRNA/miRNA1.htm

## Abbreviations

5′-UTR: 5′-untranslated region
CSCs: cancer stem cells
IRES: internal ribosome entry sites
NPC: nasopharyngeal carcinoma
NPECs: nasopharyngeal epithelial cells
PCBP: poly (rC)-binding protein
PRC1: polycomb-repressive complex 1
PTB: polypyrimidine tract-binding protein
unr: upstream of N-ras.
