# Supplementary material for: Internal Ribosome Entry Sites Mediate Cap-Independent Translation of Bmi1 in Nasopharyngeal Carcinoma
Source: Front Oncol. 2020 Sep 8;10:1678. doi: 10.3389/fonc.2020.01678 (PMC7506037; doi:10.3389/fonc.2020.01678)
Supplement: Supplementary file 1 [file Image_1.pdf]

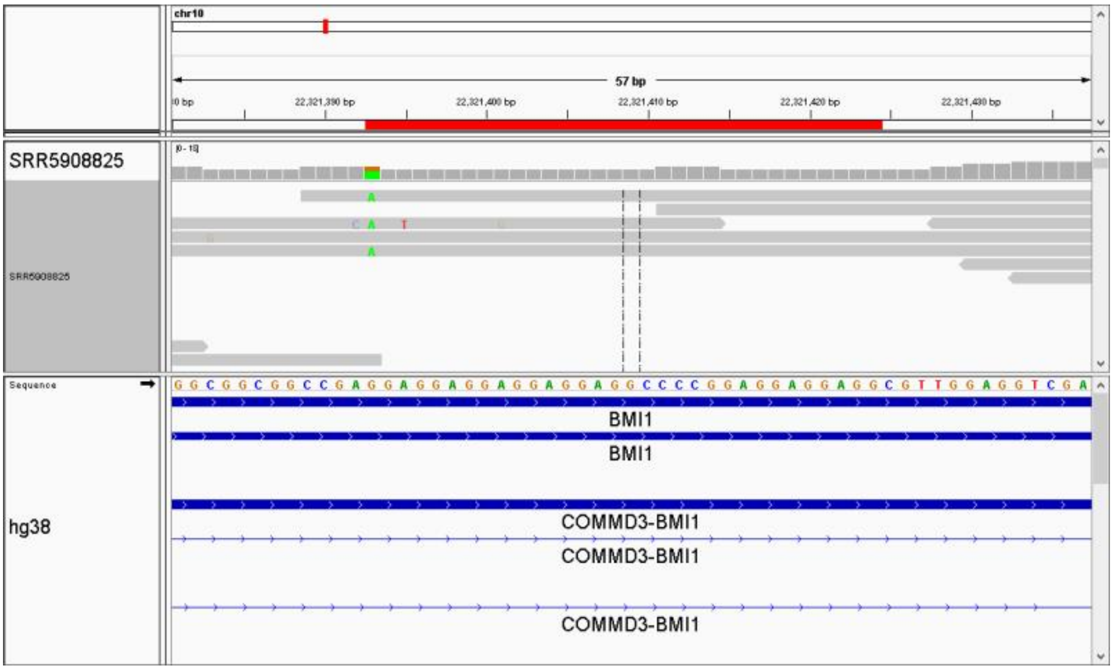

Supplementary Figure 1. There is only one patient with G/A mutation in the IRES2 element of Bmi1.

The RNA-seq data from 113 patients with nasopharyngeal carcinoma (GSE102349) was analyzed for the mutation of IRES2 in the Bmi1 5'-UTR. The G/A mutation (**GGAGGAGGAGGAGGAGGCCCCGGAGGAGGAGG ----> AGAGGAGGAGGAGGAGGCCCCGGAGGAGGAGG**) was found in IRES2 motif of the sample (SRR5908825). No mutation was found in the IRES2 motif of the rest of 112 samples. It is hard to analyze the mutation in the IRES1 element, which locates at the nearby of the transcription start sites (TSS) of Bmi1.
